# Astaxanthin Attenuates Fish Oil-Related Hepatotoxicity and Oxidative Insult in Juvenile Pacific White Shrimp (*Litopenaeus vannamei*)

**DOI:** 10.3390/md18040218

**Published:** 2020-04-17

**Authors:** Yingying Yu, Yang Liu, Peng Yin, Weiwen Zhou, Lixia Tian, Yongjian Liu, Donghui Xu, Jin Niu

**Affiliations:** 1 State Key Laboratory of Biocontrol, Institute of Aquatic Economic Animals and Guangdong Provincial Key Laboratory for Aquatic Economic Animals, School of Life Science, Sun Yat-sen University, Guangzhou 510275, China; yuyinyin1233@163.com (Y.Y.); peng.yin@hi.no (P.Y.); xszww@foxmail.com (W.Z.); Lixiatian2002@163.com (L.T.); lsslyj@mail.sysu.edu.cn (Y.L.); 2 Guangdong Key Laboratory of Animal Molecular Design and Precision Breeding, School of Life Science and Engineering, Foshan University, Foshan 528225, Guangdong, China; 17303468530@163.com; 3 Laboratory of Traditional Chinese Medicine and Marine Drugs, Department of Biochemistry, Traditional Chinese Medicine and Marine Drugs, School of Life Sciences, Sun Yat-sen University, Guangzhou 510275, China; dongxugz@163.com

**Keywords:** astaxanthin, oxidized fish oil, *Litopenaeus vannamei*, oxidative insult, hepatopancreas protection

## Abstract

The present study investigated the effect of dietary astaxanthin (AX) on the growth performance, antioxidant parameters, and repair of hepatopancreas damage in Pacific white shrimp (*Litopenaeus vannamei*). To evaluate the hepatopancreas protective function of AX in shrimps, we compared the effect of five isonitrogenous and isoenergetic diets under oxidized fish oil conditions with varying AX levels during the 50-day experimental period. The formulated diets were as follows: (i) OFO (oxidized fish oil); (ii) OFO/AX150 (oxidized fish oil + AX150 mg/kg); (iii) OFO/AX250 (oxidized fish oil + AX250 mg/kg); (iv) OFO/AX450 (oxidized fish oil + AX450 mg/kg); and, (v) control group (fresh fish oil). Results showed that the oxidized fish oil with 275.2 meq/kg peroxide value (POV) resulted in a substantial decrease in the final body weight of *L. vannamei* (*P* > 0.05) and induced some visible histopathological alterations in the hepatopancreas. Growth performance was significantly higher in shrimps fed with the OFO/AX450 diet than those fed with the OFO diet (*p* < 0.05). However, no significant difference was observed when the OFO/AX450 diet was compared to the control diet containing fresh fish oil (*p* > 0.05). Moreover, shrimps under the OFO/AX450 diet displayed a significant improvement in hepatopancreatic health and showed a reduction of malondialdehyde (MDA) compared to those under the OFO diet (*p* < 0.05). Dietary AX improved the antioxidant capacity of *L. vannamei* by increasing the catalase (CAT) activity in the hemolymph. Acute salinity change test showed a higher shrimp survival rate under OFO/AX450 diet than the OFO diet (*p* < 0.05), suggesting that AX can contribute to enhanced stress tolerance. In conclusion, our data suggest that AX confers dose-dependent protection against OFO-induced oxidative insults and hepatopancreatic damage in shrimp.

## 1. Introduction

The marine carotenoid astaxanthin (AX) is naturally found in a wide variety of aquatic organisms, such as microalgae, crustaceans (crabs, lobsters, and shrimp), and fish (salmon and trout) [1,2]. AX has a hydrophobic polyunsaturated polar structure on both ends of the conjugated olefins structure, facilitating its precise positioning within cell membranes and circulating lipoproteins. In effect, AX exhibits a potent antioxidant function as a powerful scavenger of oxygen-free radicals, so as to decrease the oxidative stress and lipid peroxidation [3]. AX is renowned as a powerful antioxidant that has been reported to surpass those of β-carotene or lutein and even α-tocopherol [4]. In crustaceans, some studies demonstrated that dietary astaxanthin could increase the total antioxidant capacity (TOC), improve growth performance [5,6,7], increase survival [8], and enhance resistance to different types of environmental stresses, including salinity stress [9], oxygen depletion stress [10] and high temperature stress [11]. Therefore, dietary astaxanthin, which is a remarkable antioxidant, could be used to improve the growth performance and enhance the stress tolerance of marine species.

Lipid peroxidation is the oxidative deterioration of polyunsaturated fatty acids (PUFAs) through a free-radical chain reaction. Lipid peroxide can damage the lipid-rich cell membranes of the organism [12]. Fish oil, which is rich in PUFAs, is necessary to maintain the cell membrane fluidity of aquatic animals. However, PUFAs are very susceptible to peroxidation to form toxic lipid hydroperoxides, the primary oxidation products, through a free radical related process. The unstable lipid hydroperoxides can decompose readily to fatty acid alkoxy [13] or break down to release a series of secondary oxidation products, such as aldehydes, ketones, alcohols, and carboxylic acids [14]. These by-products of oxidation are the primary source of unpleasant flavor and odor in decomposing marine organisms, as a result of the damage of cellular biomembranes and animal health deterioration [15,16]. Previous studies have shown that the use of oxidized oil in fish diet can delay development or survival by modulating hemoglobin concentration, and glycolytic activity, lipid peroxidation [17,18,19,20], and the deposition of α-tocopherol [21]. In contrast, our understanding of the effects of lipid oxidation on crustacean species is limited. Koshio et al. (1994) reported that oxidized oil can result in growth reduction, reduced post-larval size, and poor health in *Penaeus japonicas* [22]. Laohabanjong et al. (2009) showed that the oxidation of fish meal lipid can result in abnormalities with haemocytic infiltration, atrophy of tubular epithelial, and nodule formation in tiger shrimp *Penaeus monodon* [23]. Wang et al. (2015) found that moderate and high oil oxidization can lower the survival and feeding efficiency of juvenile Chinese mitten crab *Eriocheir sinensis* [24]. Although oxidized fish oil has a negative effect on crustaceans, only a few studies have explored how to reduce the adverse impact of lipid peroxidation through diet management.

The Pacific white shrimp (*Litopenaeus vannamei*) is the most commonly cultured crustacean in South China. In recent years, the deteriorating environments have seriously affected shrimp farming, especially in the subtropical regions of Guangdong province in China where the climate is humid and the temperature is high throughout the year [25,26]. In commercial situations, feedstuffs or diets are often stored in paper bags, making the lipids readily susceptible to oxidation. Some studies have shown that oxidative damage in aquatic animals caused by oxidative lipids can be prevented by exogenous antioxidants, such as the supplementation of vitamin E or α-tocopherol [21,24,27]. In this study, the potential benefits and protective effect of dietary AX in *L. vannamei* were evaluated in shrimps fed with oxidized fish oil (OFO) for a 50-day period. The chronic effects of dietary OFO and the role of dietary AX, as a possible chemoprotective agent against OFO, were observed. The objectives of this study were (1) to evaluate the chronic effects of dietary OFO on growth performance and hepatopancreas damage of *L.vannamei* and (2) to determine the benefits of dietary AX in preventing or ameliorating the effect of dietary OFO.

## 2. Results

### 2.1. Growth Performance and Feed Utilization

The growth performance and survival of shrimp are shown in Table 1. After the 50-day feeding trial, the final body weight (FBW) of shrimps fed with the OFO + AX450 diet was significantly higher than those fed with the OFO diet (*p* < 0.05). However, no significant difference was observed between shrimps fed with the OFO + AX450 diet and the control fresh fish oil diet (*p* > 0.05). Lowest weight gain (WG), specific growth rate (SGR), and survival rate were found in shrimps fed with the OFO diet, although no significant differences were found in WG, SGR, and survival among other dietary groups (*p* > 0.05). Similarly, a higher feed conversion ratio (FCR) was found in shrimps fed with the OFO + AX150 diet than those fed with the OFO diet and no significant differences were found among other dietary groups (*p* > 0.05).

### 2.2. Shell Astaxanthin Concentration

**Figure 1:**
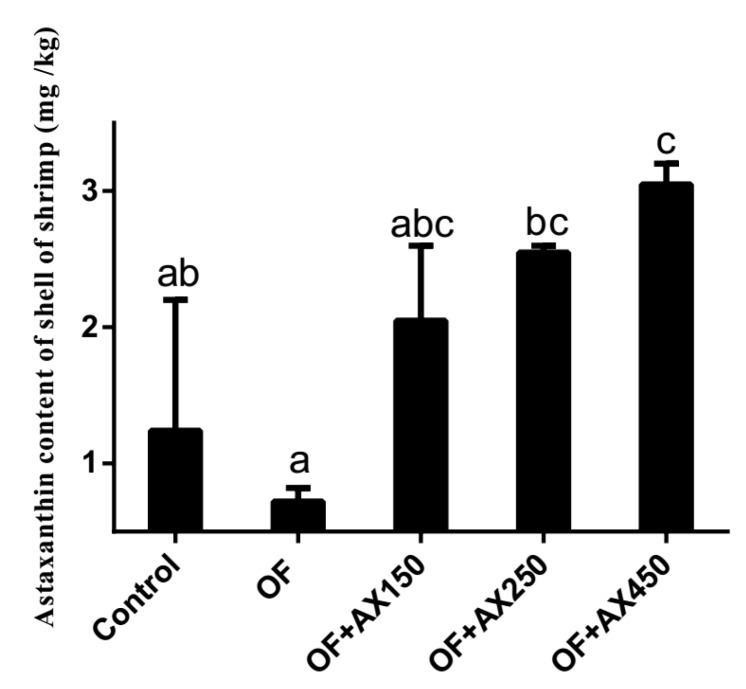
The astaxanthin content of *L. vannamei* shell fed with different diets over the feeding trial.

The effect of experimental diets on AX concentration in the shell of shrimps (% dry weight basis) is presented in Figure 1. We observed an increasing AX content in the shell when we increased the level of dietary AX. Shrimps fed with the OFO diet showed a significantly lower AX level in its shell when compared to those getting the OFO + AX250 and OFO + AX450 diets (*p* < 0.05). We observed no significant difference between shrimps fed with the control fresh fish oil and OFO + AX150 diets (*p* > 0.05).

### 2.3. Survival Rate of Shrimp after the Acute Salinity Change Test

**Figure 2:**
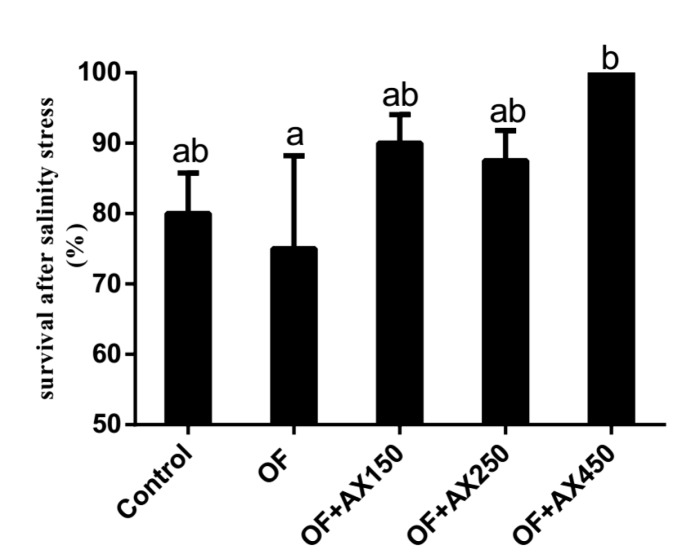
Survival rate (%) of *L.vannamei* fed control diet and expermient diets after acute salinity changes for 5 h.

The survival rates of shrimps after the acute salinity change test for 5 hours in all dietary groups are presented in Figure 2. The lowest survival rate was found in shrimps fed with the OFO diet and was significantly lower than those fed with the OFO + AX450 diet (*p* < 0.05). No significant differences were observed in shrimps fed with the control fresh fish oil, OFO + AX150, and OFO + AX250 diets (*p* > 0.05). 

### 2.4. Hepatopancreatic and Hemolymph Immune Parameters

The hemolymph and hepatopancreas immune parameters of shrimps are shown in Table 2. High hepatopancreatic and hemolymph malondialdehyde (MDA) contents in shrimp were induced by the OFO diet, thus, its hemolymph MDA content was significantly higher than in the control group (*p* < 0.05). The lowest catalase (CAT) activity of hemolymph was found in shrimps fed with the OFO diet and that CAT activity showed an increasing pattern along with the increasing dietary AX. 

### 2.5. Muscle Fatty Acids Composition

The muscle fatty acids composition of shrimps are presented in Table 3. Unsaturated fatty acid content (monounsaturated and polyunsaturated fatty acids) in the muscle of shrimps fed with the OFO diet was significantly lower than those fed with the OFO + AX250 diet (*p* < 0.05). In shrimps fed with the OFO diet, the levels of eicosapentaenoic acid (EPA), docosahexaenoic acid (DHA), and omega-3 (*n*-3) polyunsaturated fatty acids (PUFA) were the lowest, while *n*-6 PUFA was the highest.

### 2.6. Hepatopancreas Histology

**Figure 3:**
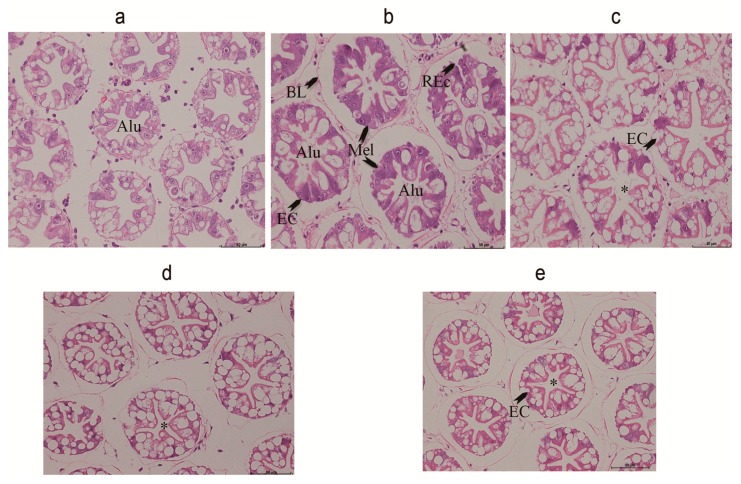
Hepatopancreas from experiment diets treated *L. vannamei*. (**a**) The hepatopancreas from a shrimp fed with contol diet for 50 d. Bar = 50 μm. Tubular epithelial cells have detached from the basal lamina with this treatment, and the tubules have atrophied, the tubular epithelial cells are slightly vacuolated. (**b**) The hepatopancreas from a shrimp fed with oxidized fish oil diet for 50 d. Bar = 50 μm. The hepatopancreas from a shrimp showing that the tubular epithelial cells are heavily vacuolated; and some are ruptured. Melanization of the epithelial cells has appeared. (**c**) The hepatopancreas from a shrimp fed with oxidized fish oil + AX 150 mg/kg diet for 50 d. Bar = 50 μm. Transverse section of the middle proximal region of tubules showing that tubules are well arranged and appear as a star shape in the lumen. Different cell types can be observed, and the quantity of these cells are more than the control and OFO groups. (**d**) The hepatopancreas from a shrimp fed with oxidized fish oil + AX 250 mg/kg diet for 50 d. Bar = 50 μm. (**e**) The hepatopancreas from a shrimp fed with oxidized fish oil + AX 450 mg/kg diet diet for 50 d. Bar = 50 μm. Both (**e**) and (**f**) show that tubules are normally appearing as a star shape in the lumen. Different cell types can also be observed. ALU, abnormal lumen; BL, basal lamina; REc, ruptured epithelial cells; Mel, melanization of cells; *, star shape of the lumenof cells; *, star shape of the lumen.

We observed lesions in the hepatopancreas of shrimps fed with the OFO diet. The tubular epithelial cells of the hepatopancreas were heavily vacuolated and some were ruptured. Melanization of the epithelial cells and significant reduction of B-cells were evident (Figure 3b). Dietary AX supplementation markedly attenuated the hepatopancreatic tissue injury induced by the oxidized fish oil, so that a similar or even better histopathological picture was observed in comparison to the control group (Figure 3a). Furthermore, increased distance between adjacent tubules and abnormal lumen of the tubules was observed in the control group (Figure 3a). AX co-treatment stored the normal electron microscopic appearance of hepatopancreas, making similar or even better histology than that observed in the control group (Figure 3c–e)

**Figure 4:**
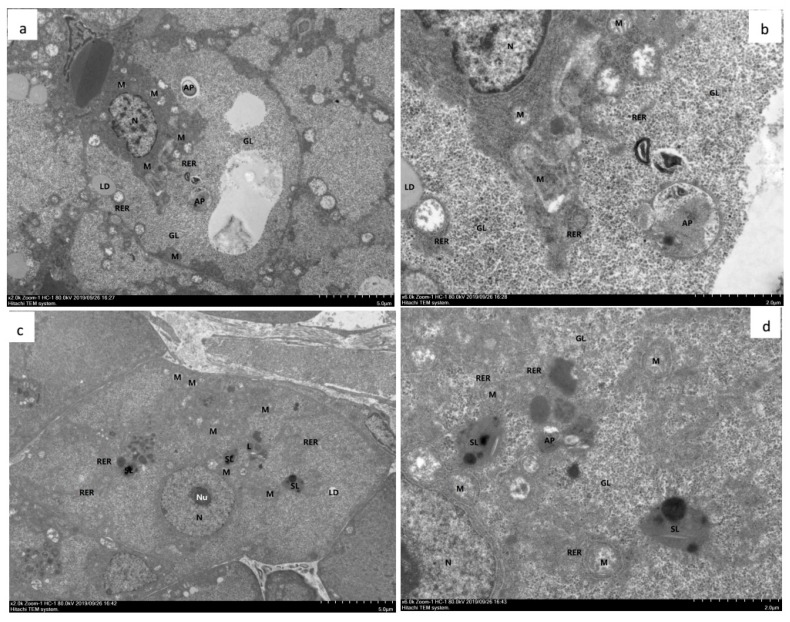
Hepatopancreas from experimental diets treated with *Litopenaeus vannamei*. **a**–**d** TEM (**a**) and (**b**). The hepatopancreas from shrimp fed with OFO diet for 50 d. The hepatocytes showed moderate edema, intact cell membrane, moderate swelling and vacuolation of organelles in the cytoplasm, more vacuoles in the cells, and local electron density decreased. The mitochondria (M) showed slight swelling, most of the mitochondrial matrix slightly weakened, the internal cristae decreased, and the cristae disappeared in severe cases; the RER structure was fuzzy. Glycogen (GL) was abundant. Autophagy (AP) was abundant. Lipid droplets (LD) are present individually. There are more lysosomes (L) and secondary lysosomes (SL). (**c**) and (**d**) The hepatopancreas from shrimp fed with OFO/AX450 diet for 56 d. The liver cells showed mild edema, intact cell membrane, slight swelling of intracellular organelles, and a few vacuolations. autophagy (AP), mitochondria (M), large vacuoles (V), nucleoli (Nu), nucleus (N), Glycogen (GL), lysosomes (L), secondary lysosomes (SL), rough endoplasmic reticulum (RER).

TEM analysis demonstrated that in the hepatopancreas from shrimp fed with OFO and OFO/AX450 diet for 50 d, the rough endoplasmic reticulum in many B cells undergoes a marked swelling and vesiculation and have showed large vacuoles, the internal cristae of mitochondria decreased, the cristae disappeared in severe cases, and the RER structure was fuzzy (Figure 4a,b). Dietary OFO/AX450 supplementation attenuated the hepatopancreatic injury induced by OFO (Figure 4c,d).

## 3. Disscussion

The present study investigated whether AX supplements confer protection against the chronic effects of the OFO diet on the growth performance and hepatotoxicity in Pacific white shrimps. Dietary OFO has been related to reduced nutrient digestibility [28] and low nutritional value of feed materials, by destroying the PUFA contents and other essential food constituents [29]. It has been shown in several studies that feeding oxidized lipids did not lead to any reduction in the growth of several different fish species [12,30,31]. On the contrary, a reduction in growth caused by OFO was also observed in some aquatic animals, including *Labeo rohita fingerlings* [32], *Pagrus major* [33] and *M. salmoide* [34]. Yang et al. (2015) demonstrated a significant reduction of growth in juvenile *L. vannamei* fed with the OFO diet (peroxide value (POV): 234.84 meq/kg) [35]. The reduced weight gains of the animals fed with the dietary oxidized lipid may be due to altered palatability, leading to poor growth performance [21,24,27].

Dietary AX, as a powerful antioxidant, could enhance nutrient utilization and ultimately improve growth. Aside from that, AX could increase stress tolerance and play an important role in the intermediary metabolism of aquatic animals [36,37,38]. In this study, adequate AX supplements in the OFO diet substantially improve the growth performance of *L. vannamei*. AX has the same function as Vitamin E [39] and Selenium (Se) [40] in protecting lipid oxidation, thereby maximizing nutrient availability in the diet of *L. vannamei* [6,10]. 

The thiobarbituric acid reactive substances (TBARS) test is widely used as a single assay for measuring lipid peroxidation by-product MDA. In this study, we show that the MDA level in the hemolymph increases significantly in shrimps fed with the OFO diet, suggesting that OFO induces a state of oxidative stress. Dietary AX abrogates the degree of lipid peroxidation, as the MDA level decreases in the hemolymphs of shrimps when AX is incorporated into the OFO diet. Similar to our study, other researchers documented that the TBARS level was increased in serum and liver due to dietary oil oxidation [21,24,27]. CAT is one of the major antioxidant enzymes responsible for scavenging reactive oxygen species (ROS) and serves as a protective mechanism to avoid tissue damage caused by free-radicals and phagocytosis [5]. We show that CAT activity in the hemolymph of shrimp fed with the OFO diet decreases significantly. This result indicates that, under the OFO diet, the capacity of shrimp to scavenge free radicals reduces dramatically. As a powerful biological antioxidant, the moderate concentration of AX in the diet could inhibit MDA accumulation and thus the observed decline in CAT activity. Previous studies demonstrated that AX had strong O^2^ quenching activity and dietary AX could relieve oxidative stress [6,10]. Our results suggest that dietary AX could partially alleviate oxidative stress caused by the OFO diet in *L. vannamei*, which could help to reduce damage caused by reactive oxygen species.

Previous studies indicated that dietary AX could enhance stress tolerance in aquatic animals. Pan et al. (2003) and Chien and Shiau (2005) showed that *P. monodon* fed with AX diets had an increased survival rate after the thermal, osmotic, ammonia, and low dissolved oxygen (DO) challenges [41,42]. Chien and Shiau (2005) suggested that under low DO conditions, *M. Japonicus* fed with algae or synthetic AX diets had longer survival time than the control group [41]. In the acute salinity change test, we show that shrimps fed with the OFO diet have a lower survival rate compared with the control group, while those fed with AX supplements have a higher survival rate compared with the OFO group. These results indicate that AX supplementation could ameliorate the low resistance capacity to salinity stress in shrimps fed OFO. 

It is well known that OFO could induce pathological changes in the hepatopancreas of terrestrial and aquatic organisms. Chen et al. (2012) suggested an increased oxidative stress in *M. salmoides* fed with an oxidized lipid may account for the observed pathological changes [43]. The hepatopancreas is an important digestive organ that possesses several functions, including absorption, digestion, storage, secretion, and detoxification in crustaceans [44,45]. The hepatopancreas is essentially composed of branched tubules and different types of epithelial cells lining the tubules. The hepatopancreas of a crustacean is sensitive to diet-born pollutants and is often used to monitor the effect of various toxicants [46]. In this study, histological examinations in shrimps under the OFO diet indicate degenerative features in the tubules and epithelial cells of the hepatopancreas, but the addition of dietary AX attenuates these histopathological changes (Figure 3). From the TEM analysis, we found that rough endoplasmic reticulum in the B cells underwent a marked swelling and vesiculation. These results suggest that shrimps could benefit from dietary AX by ameliorating any pathological changes associated with oxidized lipids.

The diets containing oxidized lipids might be considered deficient in essential fatty acids according to the National Research Council (2011) [47]. In the present study, fatty acid profiles in the muscle and AX in the shell were altered under dietary OFO and AX supplementation. The UFA of the muscle got the highest value in the OFO + AX250 diet treatment and was significantly higher than that in the OFO diet treatment. The present AX content was reduced from 1.24 mg/kg in the control diet treatment to 0.72 mg/kg in the OFO diet treatment, which may indicate that shrimps consuming AX supplements could resist oxidative stress. Correlation analysis showed that hepatopancreas MDA did not only have a highly significant positive correlation with UFA, but also had a significant negative correlation with survival rates over the feeding trial or after an acute salinity change test. This may suggest that mortality and stress tolerance were related to lipid peroxidation in hepatopancreas. Survival rates after the acute salinity change test had a significant positive correlation with AX content of shell, which indicates a direct or indirect relationship between AX and stress tolerance. The concentration of AX stored in shrimp was increased with an increasing level of dietary AX. Moreover, AX could reduce tissue lipid peroxidation and the decreased lipid peroxidation products may serve to protect muscle UFA content from the detrimental effects of oxidation. Consequently, the present research hypothesizes that AX might play an important role in the detoxification of peroxidation caused by dietary oxidized fish oil.

## 4. Materials and Methods

### 4.1. Diet Preparation

Juvenile *L. vannamei* were fed with five isonitrogenous and isolipidic diets. Diets were formulated with different levels of dietary oxidized fish oil and/or with astaxanthin (AX) (Lucantin^@^ Pink10%; BASF SE, Ludwigshafen, Germany) supplements. The dietary conditions were as follows: (i) OFO (oxidized fish oil); (ii) OFO/AX150 (oxidized fish oil + AX150 mg/kg); (iii) OFO/AX250 (oxidized fish oil + AX250 mg/kg); (iv) OFO/AX450 (oxidized fish oil + AX450 mg/kg); and, (v) control group (fresh fish oil) (Table 4). The peroxide value of oxidized fish oil was 275.2 meq/kg. Each diet was fed to a quadruplicate group of shrimp. Briefly, all the powdery ingredients were accurately weighed, thoroughly blended, and then lipids and water were added. Cold-extruded pellets (1.2 mm in diameter) were air-dried to approximately 10% moisture. The dried pellets were placed in the vacuum-packed bags and stored at −20 °C until used. A total of 40 g of each diet was sampled for biochemical composition analysis [48].

The oxidized fish oil was prepared as follows: (1) fish oil was oxidized by heating at 70 °C under vigorous aeration; (2) After 36 h, peroxide value (POV) was monitored every 8 h intervals until high oxidation level (275.2 meq/kg) was reached. Peroxide values were determined according to AOAC. Briefly, 5 g oil, 0.5 mL saturated KI solution and 30 mL solvent mixture comprising of acetic acid and chloroform (3:2) were combined. Titration was carried against 0.1 mol/L Na2S2O3, using 1% starch indicator. In the same way, we repeated this with the reagent blank test. Then, we calculated and analysed the measurement results. Calculation: *X* = [(*V* − *V*0) × *N* × 0.1269]/m (*X*: Peroxide value of the sample, %. *V*: Volume of sodium thiosulfate solution consumed by the sample, mL. *V*0: The volume of blank consumption of sodium thiosulfate solution, mL. *N*: The molar concentration of sodium thiosulfate standard solution, mol/L.) [33].

### 4.2. Shrimp and Experimental Conditions

The stocks of juvenile *L. vannamei* were supplied by Evergreen South Ocean Tech Co. Ltd, Zhan-jiang, China. Prior to the experiment, shrimps were acclimatized to the new environment by placing them in the tanks for two weeks, feeding with a commercial diet (Guangdong evergreen Group, Zhan-jiang, China), and providing with aerated recirculating filtered seawater. Afterward, shrimps (initial body weight of 0.53 g) were distributed randomly into 20 fiberglass tanks (300 L, 0.6 m^2^ bottom, 4 tanks per diet, 30 shrimps per tank). All groups were fed four times a day (at 7:00, 12:00, 17:00 and 21:00) by hand, with a total amount of approximately 9% of its body weight. To maintain a suitable culture condition, all uneaten food and feces were siphoned out throughout the 50-day experiment period. 

During the experimental period, temperature ranged from 27 to 30 °C, salinity was approximately 27%–30%, pH was 7.7–8.0, ammonia nitrogen was not more than 0.05 mg/L, and dissolved oxygen was not less than 6.5 mg/L.

### 4.3. Sample Collection 

All shrimps were fasted for 24 h before sampling. During sampling, shrimps were weighed individually and collected for further analysis. For each tank, 5 shrimps were collected randomly for shell AX content and muscle fatty acid (FA) analysis, whereas 6 shrimps were collected randomly for hemolymph and hepatopancreas samples. Hemolymph was sampled from the abdominal cavity or around the heart ventricle using a 1 mL syringe. The hemolymph samples were kept undisturbed in the refrigerator for 24 h at 4 °C and then centrifuged for 10 min (4 °C, 8000 rpm). The supernatants were used for the analysis of enzyme activity and malondialdehyde (MDA) content. Six samples of hepatopancreas were immediately placed into liquid nitrogen and stored at −80 °C. Before the analysis of enzyme activity, the hepatopancreas of each shrimp was weighed (0.5 g) individually and homogenized in 10× (w/v) phosphate buffer solution (0.1 mol L^−1^, pH 6.4) in ice-water. The homogenate was centrifuged (6000 rpm, 10 min) at 4 °C and an aliquot of the supernatant was used to determine the hepatic MDA.

### 4.4. Histopathological Studies 

The sampled hepatopancreas of three shrimps (per tank) were fixed in neutral 4% formalin and later embedded in paraffin. Tissue sections (8 μm thickness) of the hepatopancreas were stained with hematoxylin and eosin (H&E) stain and the final slides were examined under the light microscope for histopathological lesions.

For the TEM microscopy, the specimens were fixed in 2.5% glutaraldehyde with 0.1 M phosphate-buffer and postfixed in 1% OsO_4_. The specimens were emmbedded in Spurr’s medium epoxy resin after dehydration in a graded acetone (Polysciences Ltd., Warrington, PA, USA). An ultratome Leica UCT was used for cutting ultrathin sections, and then ultrathin sections were stained with lead citrate and a saturated solution of uranylacetate in 50% ethanol. Ultrathin sections were then screened with a TEM (FEI Tecnai G2 20, Holland) at 150 kV and the images were acquired by a camera Megaview III (SIS GmbH), equipped with software AnalySIS.

### 4.5. Fatty Acid Composition

Total lipid was extracted using chloroform: methanol (2:1, v/v). The capillary gas chromatography (GC) method was employed to determine the fatty acid profile. The HP6890 (FID detector; Agilent Technologies, Palo Alto, CA, USA) and SPTM-2380 column (30 m × 0.25 mm × 0.20 µm) were used on the GC machine. The separation was carried out with nitrogen as the carrier gas. Temperature of the column increased from 140 to 240 °C at 4 °C min^−1^, held at 140 °C for 5 min and at 240 °C for 10 min, with a detector at 260 °C. A split injector (50:1) at 260 °C was used. Each fatty acid was identified by the retention time from the chromatographic standard (Sigma, Northampton, UK). Peak areas were determined using the Varian software (Varian, Inc., Palo Alto, CA, USA). The concentration of the individual fatty acid was expressed in percentage of total fatty acids.

### 4.6. Astaxanthin Content of the Shell

According to the methods of Chien and Shiau (2005) [41], the shells of ten shrimps from each tank were dissected, freeze-dried, minced, and placed into a 50-mL polypropylene centrifuge tube. The solvent acetone (20 mL) was added into each tube to homogenize the mixture (Polytron PT-MR-3000; PT. Hartono IstanaTeknologi, Indonesia) at 12700× *g* for 1 min and then centrifuged (Hitachi 18 PR-52; Hitachi Ltd., Tokyo, Japan) at 12700× *g* for 5 min. The pellet was resuspended and centrifuged with an additional 20 mL of acetone, until the acetone extract was clear. The pooled acetone extracts were transferred into a 250-mL separatory funnel partitioned with 30 mL n-hexane and washed three times with 10% NaCl to remove the residual acetone. Then, the extract was reduced to 10 mL using a rotary evaporator. Afterward, the extract was filtered through a 0.2 Am Millipore filter and stored in three 2-mL brown vials. AX contents were determined using high-performance liquid chromatography (Agilent 1200; Agilent technologies, Waldbronn, Germany). The standard of the chromatographically pure AX was purchased from Sigma-Aldrich Co. LLC (St. Louis, MO, USA). The HPLC condition was adjusted according to the previous method of Yuan et al. (1996) [49]. Chromatographic peaks were identified by comparing retention times against known standards. 

### 4.7. Lipid Peroxidation and Enzyme Activity Assays

Lipid peroxidation (LPO) was assessed by measuring thiobarbituric acid-reactive substances (TBARS) during an acid heating reaction, as previously described of Esterbauer and Cheeseman (1990) [50]. The determination of LPO content in shrimp was carried out using the MDA detection kit (A003-1, Jian-cheng Bioengineering Institute, Nan jing, China). The results were expressed in nmol MDA equivalents per milligram protein of hepatopancreas and mmol MDA equivalents per liter of hemolymph. 

Catalase (CAT) activity was determined using a test kit (Jian-cheng Institute of Biotechnology, Nan jing, China). CAT activities of tissue was measured spectrophotometrically at 405 using a SpectraMax M5 microplate reader (Molecular Devices, Minneapolis, MN, USA). One unit of CAT activity was defined as the amount of enzyme that catalyzed the decomposition of 1.0 μmol of H_2_O_2_ per min. The results were expressed in U/L of hemolymph [48].

iNOS activity was determined using an iNOS assay kit (Jiancheng Bioengineering Institute, Nanjing, China). Briefly, 10% brain homogenate (30 μL) or serum (15 μL) was mixed with the working solution supplied in the kit, and after incubation at 37 °C for 15 min, the reaction was terminated by adding the terminating solution provided in the kit. The absorbance at 530 nm was recorded on a Hitachi U-2010 spectrophotometer (Tokyo, Japan), and iNOS activity was calculated in the reference of a standard curve. The amount of NO generated in samples was determined by measuring the absorbance at 550 nm.

### 4.8. Acute Salinity Change Experiment

A total of 10 shrimps per tank were randomly selected for the acute salinity change experiment. The test involved an immediate salinity decreasing level from 29% to 10% by adding de-chlorinated freshwater. Shrimps were carefully monitored and mortality was recorded during the whole 5-hour-experiment. 

### 4.9. Calculations and Statistical Analysis

The parameters were calculated as follows:

Percentage weight gain (WG, %) = 100 × (*Wt* − *Wi*)/*Wi*

Specific growth rate (SGR, % day^−1^) = 100 × (Ln *Wt* − Ln *Wi*)/*t*

Feed conversion rate (FCR) = feed consumed (g, dry weight)/weight gain (g, wet weight)

Survival (%) = 100 × (final amount of shrimp)/(initial amount of shrimp)

where *Wt* is the final body weight (g), *Wi* is the initial body weight (g), and *t* is the experimental duration in days.

Analyses of all data included the homogeneity test, and if similar variances were observed, a one-way ANOVA was performed to determine the main effect of dietary manipulation. When significant differences (*P ≤* 0.05) emerged after one-way ANOVA, the group means were compared further using Duncan’s multiple range test. On the other hand, if the data did not have similar variances, the non-parametric Kruskal–Wallis test was applied, followed by pairwise multiple comparisons if the results of the Kruskal–Wallis test showed significant difference (*P* ≤ 0.05). Correlation analyses were also used to determine the relationships between biochemical parameters. All data were analyzed using the SPSS 19.0 software (SPSS, Chicago, IL, USA) and the results were presented as the means ± SEM.

## 5. Conclusions

Overall, the present work demonstrated that dietary oxidized fish oil (POV: 275.2 meq/kg) could cause obvious histopathological changes and oxidative stress in the *L. vannamei*. Moreover, dietary AX may ameliorate these effects by increasing the CAT activity and decreasing MDA accumulation in the hemolymph of shrimp. Shrimps fed with dietary AX had a better capability to sustain the acute salinity stress tolerance test. More in-depth studies are required to verify the potential mechanism of AX in alleviating oxidative insult and hepatotoxicity in shrimps and other crustaceans.

## Figures and Tables

**Table 1:** Growth performance and feed utilization of the juvenile *L. vannamei*, fed with different diets over the 50-day feeding trial.

Items	D1	D2	D3	D4	D5
	Control	OFO	OFO + AX150	OFO + AX250	OFO + AX450
Initial body weight (g)	0.53 ± 0.02	0.53 ± 0.01	0.53 ± 0.01	0.53 ± 0.02	0.53 ± 0.01
Final body weight (g)	9.62 ± 1.42 ^a,b^	8.31 ± 0.33 ^a^	8.78 ± 0.23 ^a,b^	10.46 ± 1.77 ^a,b^	11.09 ± 2.13 ^b^
Weight gain (%)	1674.2 ± 106.8 ^a,b^	1457.7 ± 62.2 ^a^	1548.7 ± 24.2 ^a,b^	1866.7 ± 335.9 ^a,b^	1980.8 ± 399.9 ^b^
SGR (% day^−1^)	5.13 ± 0.10	4.90 ± 0.04	5.00 ± 0.03	5.30 ± 0.17	5.40 ± 021
Survival (%)	87.78 ± 7.78	75.83 ± 6.85	81.11 ± 6.76	88.89 ± 9.50	86.67 ± 8.39
FCR	1.37 ± 0.07	1.42 ± 0.13	1.48 ± 0.02	1.18 ± 0.12	1.21 ± 0.26

^a,b^ Mean values within a row without a common superscript letter were significantly different (*p* ≤ 0.05). Data represent mean ± SEM of four replicates.

**Table 2:** Several hemolymph and hepatopancreas parameters of the juvenile *L. vannamei* fed with different diets.

Items	D1	D2	D3	D4	D5
	Control	Oxidized Fish Oil (OFO)	OFO + AX150	OFO + AX250	OFO + AX450
Hemolymph malondialdehyde (MDA) (mmol L^−1^)	4.40 ± 0.40 ^a^	6.76 ± 0.75 ^b^	4.78 ± 0.29 ^a,b^	5.22 ± 0.22 ^a,b^	6.01 ± 0.36 ^a,b^
Hemolymph catalase (CAT) (U L^−1^)	1.07 ± 0.06 ^b,c^	0.54 ± 0.26 ^a^	0.90 ± 0.19 ^a,b^	1.17 ± 0.14 ^b,c^	1.50 ± 0.13 ^c^
Hepatopancreas MDA (nmol mg^−1^prot )	1.96 ± 0.50	3.32 ± 1.10	3.06 ± 0.81	2.11 ± 0.15	1.97 ± 0.12
Hepatopancreas iNOS (U mg^−1^prot)	1.35 ± 0.11 ^a,b^	1.28 ± 0.09 ^a^	1.39 ± 0.10 ^a,b^	1.54 ± 0.15 ^a,b^	1.62 ± 0.10 ^b^

^a,b,c^ Mean values within a row without a common superscript letter were significantly different (*p* ≤ 0.05). Data represent mean ± SEM of four replicates.

**Table 3:** Fatty acid composition of muscle from the juvenile *L. vannamei* (weight %).

Metabolite ^a.^	D1	D2	D3	D4	D5
14:0	0.17 ± 0.03	0.33 ± 0.14	0.23 ± 0.04	0.19 ± 0.05	0.19 ± 0.04
15:0	0.18 ± 0.02	0.19 ± 0.01	0.20 ± 0.00	0.17 ± 0.02	0.18 ± 0.01
16:0	16.83 ± 1.23	18.60 ± 1.90	18.27 ± 0.55	15.10 ± 1.5	16.03 ± 1.09
16:1	1.05 ± 0.15	1.86 ± 0.66	0.98 ± 0.17	1.06 ± 0.28	0.80 ± 0.11
17:0	0.94 ± 0.02	0.93 ± 0.02	0.95 ± 0.02	0.90 ± 0.01	0.95 ± 0.02
17:1	0.71 ± 0.21	0.45 ± 0.15	0.75 ± 0.09	0.87 ± 0.23	0.68 ± 0.11
18:0	12.07 ± 0.47	11.20 ± 0.79	12.00 ± 0.38	12.17 ± 0.39	12.63 ± 0.34
18:1	20.27 ± 0.78	20.30 ± 0.90	20.63 ± 0.59	19.67 ± 1.60	20.03 ± 0.87
18:2 *n*-6	10.80 ± 0.78	11.23 ± 0.62	10.97 ± 0.28	10.50 ± 0.46	10.25 ± 0.46
18:3 *n*-3	0.58 ± 0.03	0.59 ± 0.03	0.55 ± 0.06	0.52 ± 0.02	0.54 ± 0.01
20:0	0.22 ± 0.03	0.18 ± 0.06	0.25 ± 0.04	0.18 ± 0.09	0.27 ± 0.12
20:1	1.04 ± 0.08	0.98 ± 0.12	0.96 ± 0.04	1.06 ± 0.04	1.10 ± 0.00
C20:2	1.77 ± 0.17	1.47 ± 0.31	1.77 ± 0.09	1.97 ± 0.12	1.98 ± 0.08
C22:0	0.30 ± 0.02	0.33 ± 0.04	0.30 ± 0.02	0.25 ± 0.13	0.33 ± 0.03
C 20:4 *n*-6	3.03 ± 0.30	2.68 ± 0.54	3.13 ± 0.12	3.70 ± 0.40	3.33 ± 0.19
C 20:5 *n*-3	12.97 ± 1.03	9.98 ± 0.79	12.00 ± 0.38	12.17 ± 0.39	12.63 ± 0.34
C24:0	0.37 ± 0.04 ^a,b^	0.26 ± 0.01 ^a^	0.40 ± 0.05 ^a,b^	0.54 ± 0.07 ^b^	0.45 ± 0.07 ^a,b^
C24:1	0.66 ± 0.33	0.37 ± 0.22	0.49 ± 0.19	0.95 ± 0.53	0.85 ± 0.36
C 22:5 *n*-6 (EPA)	1.63 ± 0.12	1.18 ± 0.40	1.17 ± 0.12	1.53 ± 0.32	1.65 ± 0.14
C 22:6 *n*-3 (DHA)	14.27 ± 0.64	13.47 ± 0.75	13.47 ± 0.87	14.17 ± 0.69	14.80 ± 0.83
EPA+DHA	27.23 ± 1.55	25.73 ± 1.94	25.80 ± 1.20	28.73 ± 2.36	28.18 ± 1.55
DHA/EPA	1.11 ± 0.06	1.10 ± 0.03	1.09 ± 0.04	0.99 ± 0.07	1.11 ± 0.03
SAFA ^1^	31.10 ± 0.85	31.72 ± 0.94	32.60 ± 0.90	29.44 ± 0.90	31.00 ± 1.16
UFA ^2^	68.76 ± 0.87 ^a,b^	66.11 ± 2.08 ^a^	66.88 ± 1.20 ^a,b^	70.56 ± 0.89 ^b^	68.92 ± 1.16 ^a,b^
*n*-3 PUFA ^3^	29.44 ± 1.56	22.69 ± 5.37	27.52 ± 1.16	30.79 ± 2.62	30.37 ± 1.63
*n*-6 PUFA	13.83 ± 0.49	15.08 ± 0.73	14.10 ± 0.29	14.20 ± 0.17	13.58 ± 0.39
*n*-3/*n*-6 ratio	2.14 ± 0.18	1.56 ± 0.41	1.95 ± 0.05	2.17 ± 0.20	2.24 ± 0.14

^a,b^ Mean values within a row without a common superscript letter were significantly different (*p* ≤ 0.05). ^1^ SAFA: saturated fatty acid; ^2^ UFA: unsaturated fatty acid; ^3^ PUFA: poly unsaturated fatty acid.

**Table 4:** Composition and nutrient levels of experimental diets.

Items	D1	D2	D3	D4	D5
	Control	OFO	OFO + AX150	OFO + AX250	OFO + AX450
**Ingredients (g kg^−1^ diet)**					
Fish meal	220	220	220	220	220
Soybean meal	210	210	210	210	210
Wheat flour	245	245	245	245	245
Peanut meal	100	100	100	100	100
soybean protein concentrate	60	60	60	60	60
Beer yeast	50	50	50	50	50
Chicken meal	30	30	30	30	30
Fresh Fish oil	30	0	0	0	0
Oxidized fish oil	0	30	30	30	30
Soya lecithin	10	10	10	10	10
Vitamin premix ^a^	10	10	10	10	10
Mineral premix ^b^	10	10	10	10	10
Ca(H_2_PO_4_)_2_–H_2_O	20	20	20	20	20
Vitamin C	1	1	1	1	1
Choline chloride (50%)	2	2	2	2	2
Astaxanthin (10%)	0	0	0.15	0.25	0.45
Cellulose	2	2	1.85	1.75	1.55
**Proximate analysis (g kg^−1^ diet )**					
Crude protein	390.1	391.1	392.6	392.6	393.1
Crude lipid	71	70	71	71	74
Astaxanthin (mg/kg)	0.47	0	57	170	289

^a^ Vitamin mixture (mg or g kg^−1^): vitamin A, 250,000 IU; riboflavin, 750 mg; pyridoxine HCL, 400 mg; cyanocobalamin, 1 mg; thiamin, 250 mg; menadione, 250 mg; folic acid, 125 mg; biotin, 10 mg; α-tocopherol, 2.5 g; myo-inositol, 8000 mg; calcium pantothenate, 1250 mg; nicotinic acid, 2000 mg; choline chloride, 8000 mg; vitamin D3, 45,000 IU; vitamin C, 7000 mg. ^b^ Mineral mix (g kg^−1^): ZnSO_4_·7H_2_O, 0.04 g; CaCO_3_, 37.9 g; KCl, 5.3 g; KI, 0.04 g; NaCl, 2.6 g; CuSO_4_·5H_2_O, 0.02 g; CoSO_4_·7H_2_O, 0.02 g; FeSO_4_·7H_2_O, 0.9 g; MnSO_4_·H_2_O, 0.03 g; MgSO_4_·7H_2_O, 3.5 g; Ca(HPO_4_)2·2H_2_O, 9.8 g.
